# A simplified subnormothermic machine perfusion system restores ischemically damaged liver grafts in a rat model of orthotopic liver transplantation

**DOI:** 10.1186/2047-1440-1-6

**Published:** 2012-05-09

**Authors:** Tim A Berendsen, Bote G Bruinsma, Jungwoo Lee, Vincent D’Andrea, Qiang Liu, Maria-Louisa Izamis, Korkut Uygun, Martin L Yarmush

**Affiliations:** 1 Center for Engineering in Medicine/Surgical Services, Massachusetts General Hospital, Harvard Medical School, and Shriners Burns Hospital, 51 Blossom Street, Boston, MA, 02114, USA; 2 Department of Biomedical Engineering, Rutgers University, 599 Taylor Road, Piscataway, NJ, 08854, USA

**Keywords:** Liver, Transplantation, Rat, Machine, Perfusion, Preservation, Ischemia, Donor, Shortage

## Abstract

**Background:**

Liver donor shortages stimulate the development of strategies that incorporate damaged organs into the donor pool. Herein we present a simplified machine perfusion system without the need for oxygen carriers or temperature control, which we validated in a model of orthotopic liver transplantation.

**Methods:**

Rat livers were procured and subnormothermically perfused with supplemented Williams E medium for 3 hours, then transplanted into healthy recipients (Fresh-SNMP group). Outcome was compared with static cold stored organs (UW-Control group). In addition, a rat liver model of donation after cardiac death was adapted using a 60-minute warm ischemic period, after which the grafts were either transplanted directly (WI group) or subnormothermically perfused and transplanted (WI-SNMP group).

**Results:**

One-month survival was 100% in the Fresh-SNMP and UW-Control groups, 83.3% in the WI-SNMP group and 0% in the WI group. Clinical parameters, postoperative blood work and histology did not differ significantly between survivors.

**Conclusion:**

This work demonstrates for the first time in an orthotopic transplantation model that ischemically damaged livers can be regenerated effectively using practical subnormothermic machine perfusion without oxygen carriers.

## Background

The discrepancy between the amount of livers transplanted annually and patients on the waiting list has been growing over the past decade. In 2009, the waiting list mortality rate was 15% in the United States (approximately 1,500 patients), whereas the transplantation rate was about 38% (5,000 transplantations) [1]. This shortage has driven scientific efforts to increase organ availability targeted at upgrading conventional storage methods (static cold storage (SCS) in University of Wisconsin (UW) solution) or employing graft optimization to allow for extension of donor criteria. First reported in 1967 [2], machine perfusion is a modality whereby organs can be assessed, preserved and treated. In 2009, the first clinical trial with hypothermic machine perfusion (HMP) of human livers was conducted [3]. Besides preserving organs [4], HMP can regenerate ischemic damage and shows potential for reclaiming livers derived from donation after cardiac death (DCD) [5,6]. Although an environment of hypothermia facilitates preservation by slowing cellular metabolism and thereby reducing the need for nutrients and oxygen, the low temperature can cause damage to the microvasculature. Intravital microscopy of hypothermically perfused rat livers has demonstrated that temperature-dependent cellular deformation of sinusoidal endothelial cells obstructs flow, which congests the sinusoid and decreases parenchymal perfusion [4,7]. In addition, the lower temperature decreases the fluidity of the plasma membrane, which can lead to cell lysis and increased enzyme leakage [8]. It follows that because HMP is superior to SCS at temperatures where metabolism is slowed, a separate, perhaps mechanistic phenomenon contributes to this advantage [9,10]. One possibility is that during machine perfusion, harmful waste products are flushed out of the organ and the continuous flow facilitates better availability of the beneficial solution to the peripheral parenchyma of the organ. In addition to HMP, researchers in many studies have explored normothermic machine perfusion (NMP) [11-17]. Because higher temperature involves increased metabolism, it becomes more essential to promote oxygen delivery, cellular function and bile homeostasis while restricting adverse processes such as nutrient depletion, apoptosis and cellular swelling. Raising the temperature extends both the potential for resuscitation and the risk of organ damage [11,13,14]. Although few studies have compared HMP with NMP in the same setting, NMP has been successful in regenerating warm ischemic grafts and preserving livers in rat and porcine models, proving its superiority over SCS [17-24].

Studies of machine perfusion have led to the need for additional research, but new trials have been slow to emerge. A potential reason lies in the relative complexity of machine perfusion, which also has hampered its progress when introduced in kidney transplantation [25,26]. A simplified machine perfusion protocol might expedite its implementation, benefiting from the practicality of cold storage while not relinquishing the advantages of machine perfusion. In this context, subnormothermic machine perfusion (SNMP) may offer multiple advantages: no temperature control and a moderate rate of metabolism in which beneficial processes still occur while adverse cellular processes may be controlled.

Coupled with metabolism at the temperatures used in SNMP and NMP is the need for aerobic respiration. Although erythrocytes or artificial oxygen carriers added to the perfusate are effective in delivering oxygen to the liver [11,18,27,28], Vairetti *et al*. successfully used oxygenated SNMP without an oxygen carrier to investigate the temperature-dependence and functional integrity of the graft postperfusion [29,30]. Recently, Gringeri *et al*. applied SNMP without an oxygen carrier to liver grafts for 120 minutes in a porcine model of autotransplantation [31]. Moreover, Tolboom *et al*. calculated the oxygen use of livers during machine perfusion at 20°C and 30°C with an oxygen carrier and concluded that though livers consume more oxygen at 37°C, the addition of the oxygen carrier was not required for adequate liver metabolism at room temperature [32]. Currently, no studies are underway to investigate the validity of SNMP without an oxygen carrier in a long-term transplantation survival model. Moreover, it has not been shown whether, similarly to HMP [3,8,33] and NMP [13,15,16], ischemically damaged organs can be reclaimed and successfully transplanted using this technique.

In this study, we used a SNMP protocol that did not involve dialysis, oxygen carriers or donor pretreatments such as anticoagulants or preconditioning, enabling high-fidelity simulation of DCD. We report herein for the first time that both fresh and warm ischemic livers can be successfully treated by using such a system and transplanted into healthy recipients with good 30-day survival.

## Methods

### Laboratory animals

Male Lewis rats weighing 250 to 300 g (Charles River Laboratories, Boston, MA, USA) were used for transplantation. The animals were maintained in accordance with National Research Council guidelines, and the experimental protocols were approved by the Subcommittee on Research Animal Care, Committee on Research, Massachusetts General Hospital (Boston, MA, USA). The animals were divided into four groups: the UW-Control group (*n* = 4, 3 hours at 4°C, SCS preservation and subsequent transplantation), the WI group (*n* = 4, 60 minutes at 34°C, warm ischemia and transplantation), the Fresh-SNMP group (*n* = 6, 3 hours of SNMP and transplantation) and the WI-SNMP group (*n* = 6, 60 minutes at 34°C, warm ischemia followed by 3 hours of SNMP and then transplantation).

### Liver procurement (all groups)

All surgical procedures were performed under aseptic conditions. Each animal was weighed and anesthetized with isoflurane (Baxter, Deerfield, IL, USA). The right phrenic vein was ligated, and the liver was freed of its surrounding ligaments. The infrahepatic vena cava (IHVC) was mobilized and elongated by ligation and dissection of the adrenal vein, lumbar plexus and right renal vein. The bile duct was cannulated (SURFLO 28-gauge polyethylene stent; Terumo Medical Corp, Somerset, NJ, USA) and dissected. The portal vein (PV) was mobilized, and its most proximal side vessels (gastroduodenal and splenic veins) were ligated and cut. The hepatic artery was ligated and dissected. The PV and IHVC were cross-clamped, marking the start of ischemic time, and the liver was excised from its recess and weighed. Cuffs fashioned from 16- and 14-gauge intravenous catheters (Becton Dickinson, Franklin Lakes, NJ, USA) were applied to the PV and IHVC as described by Delrivière *et al*. [34]. The suprahepatic vena cava (SHVC) was tailored for a sutured anastomosis.

### Static cold storage group

SCS was performed using UW solution (CoStorSol; Preservation Solutions, Inc, Elkhorn, WI, USA). No other preservatives, pharmaceuticals or anticoagulants were used during SCS. Rat livers were procured, prepared for transplantation and cold-stored for 3 hours (matching the established machine perfusion time), then transplanted as described below.

### DCD model (WI group and WI-SNMP group)

After procurement, the liver was placed without flushing into a chamber filled with saline and maintained at 34.0 ± 0.1°C for 60 minutes. *Ex vivo* warm ischemia has the advantage of temperature control and has been shown to be a relevant and severe model of warm ischemia [35].

### Subnormothermic machine perfusion (Fresh-SNMP group and WI-SNMP group)

**Figure 1:**
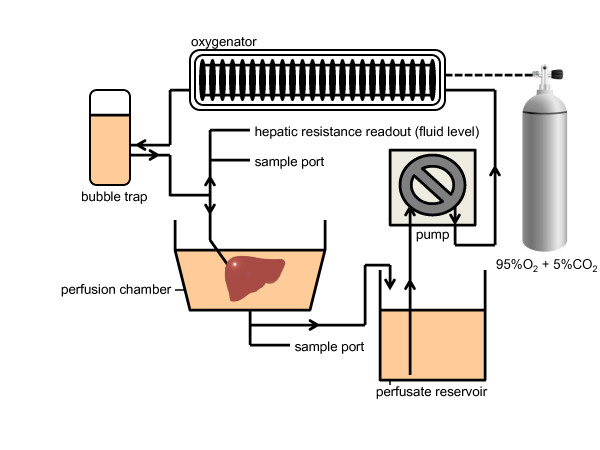
Schematic of the subnormothermic machine perfusion system.

Machine perfusion took place in a circuit that consisted of a perfusion chamber, a peristaltic pump, a membrane oxygenator and a bubble trap (Figure 1). The liver was perfused through an 18-gauge intravenous catheter (Becton Dickinson) that was connected to the PV cuff. Further details of the perfusion system and technique can be found elsewhere [36]. Temperature within the system was uncontrolled and constantly measured at 21.0°C. The total perfusate volume was 350 ml and consisted of Williams Medium E (Sigma-Aldrich, St Louis, MO, USA) supplemented with insulin (2 U/L Humulin; Eli Lilly & Co, Indianapolis, IN, USA), penicillin (40,000 U/L)/streptomycin (40,000 μg/L) (Gibco/Invitrogen, Camarillo, CA, USA), L-glutamine (0.292 g/L; Gibco/Invitrogen), hydrocortisone (10 mg/L SOLU-CORTEF; Pharmacia & Upjohn/Pfizer, New York, NY, USA) and heparin (1,000 U/L APP pharmaceuticals, Schaumberg, IL, USA). The oxygenator used a mixture of 95% O_2_ and 5% CO_2_, with the CO_2_ serving as a buffering agent, which minimized fluctuation in the perfusate pH (data not shown). Flow rate was started at 8.0 ml/minute and was adjusted according to the portal resistance, which was kept constant between 50 and 100 mM H_2_O. The total perfusion time chosen was 180 minutes, in concurrence with ATP content recovery of the grafts as detailed below, and in an effort to minimize the risk of graft contamination while providing sufficient organ recovery for transplantation.

### Perfusate analysis

Perfusate samples collected every 5 minutes for the first 20 minutes and on the half hour thereafter were tested for alanine aminotransferase (ALT) and aspartate aminotransferase (AST) using Infinity^TM^ ALT(GPT) and AST(GOT) liquid stable reagent kits (Cellomics/Thermo Electron, Pittsburgh, PA, USA). Every 30 minutes PV inflow and inferior vena cava (IHVC) outflow were sampled and analyzed using a Bayer Rapidlab 845 blood gas analyzer (Siemens Medical Solutions, Malvern, PA, USA) to record the partial pressure of oxygen (pO_2_). Those values were used to calculate the oxygen uptake rate (OUR) as detailed previously [18]. Bile was collected, quantified and reentered into the system.

### Tissue ATP content

Livers (*n* = 3 per time point) were flash-frozen in liquid nitrogen directly after procurement (Fresh-SNMP group), after 60 minutes of warm ischemia (WI group/WI-SNMP group) and after 1, 2 and 3 hours of SNMP (Fresh-SNMP group/WI-SNMP group). Various sections (*n* = 7 ± 1) were sampled per liver. Cellular ATP levels were measured using the ApoSENSOR ATP Luminescence Assay Kit (BioVision Inc, Milpitas, CA, USA). The results were normalized for protein content using a Bradford assay (Fisher Scientific, Pittsburgh, PA, USA).

### Orthotopic liver transplantation

Surgery was performed by TB in all cases according to the technique invented by Kamada [37] and described in detail by Delrivière *et al*. [34]. During the final 20 minutes of perfusion, the recipient hepatectomy was carried out up to the start of the anhepatic phase. At the end of machine perfusion, the liver was weighed and flushed with sterile PBS containing 10 U/ml heparin. The recipient hepatectomy was completed, and the donor graft was transplanted, starting with the SHVC anastomosis using a Prolene 7–0 polypropylene suture (Ethicon, Inc, Somerville, NJ, USA). Portal reperfusion occurred 13 to 17 minutes after the start of anhepatic time and approximately 20 to 30 minutes after the end of machine perfusion. Anastomoses of the IVC and bile duct were completed, the animal was administered 1 to 2 ml of sterile PBS intravenously and the abdomen was irrigated with warm PBS and closed. The skin was closed, and the animal was placed under a heat lamp to recover.

### Posttransplantation analysis

Blood samples (about 80 μl) were taken from transplantation recipients by way of tail-snip hourly after the procedure for 3 hours, then daily for 7 days after the surgery and finally after 30 days. The blood samples were analyzed using a Piccolo xpress blood chemistry analyzer and a metabolic panel (Abaxis North America, Union City, CA, USA). The animals were inspected regularly for signs of jaundice or infection and were killed 30 days posttransplantation. The liver was mobilized, resected and prepared for histology.

### Histology

Livers from each group were sacrificed for histological analysis. Samples were incubated overnight in formalin and embedded in paraffin, then processed and stained with H & E and terminal deoxynucleotidyltransferase 2′-deoxyuridine 5′-triphosphate nick-end labeling (TUNEL). As a negative control for cellular damage, stains derived from fresh liver tissue were used, in comparison to samples from the preserved, treated and transplanted groups. As a positive control for apoptosis, the TUNEL stains from the WI group were used because it has been reported that tissue from warm ischemic liver can exhibit extensive cellular degeneration and apoptosis [16,38,39].

### Statistical analysis

A paired Student’s *t*-test was applied to compare values for each parameter independently. The results were deemed statistically significant if time point comparisons had a *P*-value <0.05. Statistical significance is indicated in each figure where applicable.

## Results

### Survival

**Figure 2:**
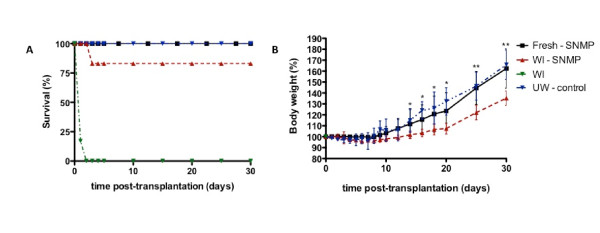
**Follow-up of recipients posttransplantation.(A)** Survival rates of subnormothermically perfused fresh and warm ischemic (WI) livers, as well as the simple cold storage (SCS) control group and the nonperfused WI group. **(B)** Body weights are normalized to preoperative weights. Error bars = SD. *P < 0.05 between WI-SNMP and UW-Control groups. **P < 0.05 between WI-SNMP and both the Fresh-SNMP and UW-Control groups.

Survival data are shown in Figure 2A. All recipients from the UW-Control and Fresh-SNMP groups survived the 30-day observation period. In the WI-SNMP group, one animal died after approximately 72 hours and the remaining five animals survived the 30-day period. All animals in the WI-only group died within 24 hours after transplantation. Body weight trends are shown in Figure 2B. After an initial drop postoperatively, weight gain resumed 7 to 14 days after transplantation in all survivors. Liver recipients in the WI-SNMP group show a significantly delayed recovery in body weight.

### Subnormothermic machine perfusion

**Figure 3:**
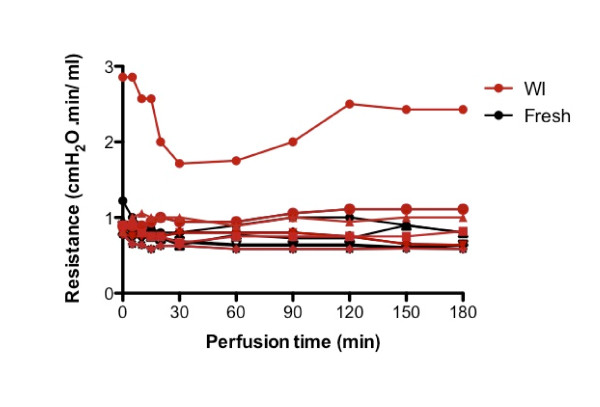
**Hepatic resistance (portal pressure/flow rate) during subnormothermic machine perfusion (SNMP) of fresh and warm ischemic groups.** Each line indicates a separate SNMP experiment (*n* = 6 per group).

Physiological flow rates in the PV were determined previously at 1.7 to 2.3 ml/minute/g liver [40]. In our system, portal flow rates were based on data from previous experiments [32,41] and ranged from 0.8 to 1.2 ml/minute/g liver. The flow rate was adjusted to the portal pressure, with the objective of keeping the portal pressure constant. The hepatic vascular resistance is expressed as a function of the portal flow and pressure in Figure 3. It did not differ significantly between any of the livers, with the exception of one liver (WI-SNMP group) that showed markedly increased vascular resistance throughout perfusion (Figure 3). The recipient of this particular liver died on the third day posttransplantation, the only nonsurvivor in the WI-SNMP group.

**Figure 4:**
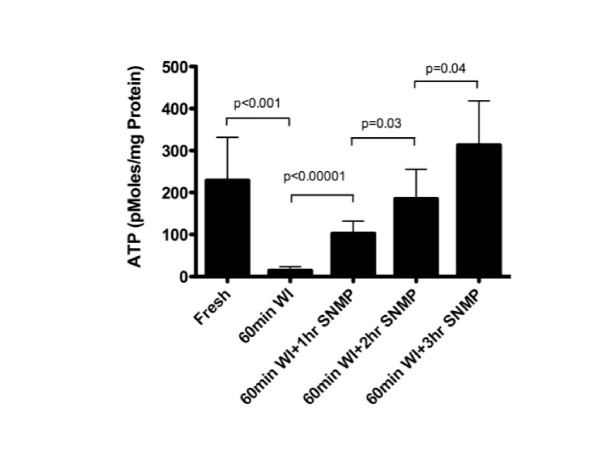
**ATP analysis of fresh, warm ischemic and subnormothermically perfused livers.** Error bars = 95% CI.

ATP analysis was performed to assess energetic depletion during WI and recovery during SNMP (Figure 4). After 60 minutes of warm ischemia, the average ATP content diminished to about 6% of fresh levels. This level was restored after approximately 2.5 hours of SNMP, with ATP values eventually exceeding the fresh ATP level.

**Figure 5:**
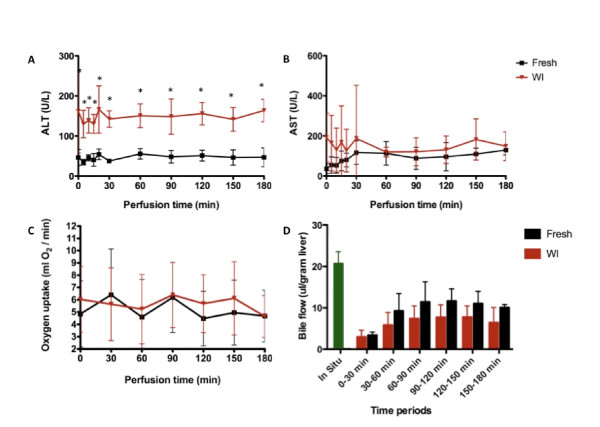
**Parameters studied during subnormothermic machine perfusion of fresh and warm ischemic livers.** Subnormothermic machine perfusion of fresh and warm ischemic (WI) livers showing levels of alanine aminotransferase **(A)**, aspartate aminotransferase **(B)**, oxygen uptake **(C)** and bile production **(D)**. The *in situ* bars represent 30 minutes of bile collection *in situ* (in the 1-hour WI group, this collection predates the WI treatment). Error bars = SD. ^*^P < 0.05 between fresh and WI groups.

During SNMP, the perfusate was analyzed for hepatocyte damage indicators. Figures 5A and 5B show cumulative levels of ALT and AST. The levels of ALT, but not of AST, were consistently higher in the WI-SNMP group. Because these data are cumulative, the horizontal trend in the graph indicates stabilization of transaminase output in the perfusate.

The pO_2_ in the perfusate was used to calculate the OUR (Figure 5C). The OUR was stable during SNMP, without differences between the groups. The pO_2_ in the outflow remained above 200 mmHg, indicating that the pO_2_ in the inflow perfusate was sufficient for hepatic oxygenation.

As a determinant of synthetic function, bile production is a prerequisite for successful transplantation [42]. *In vivo* bile flow ranges from 15 to 20 μl/g liver in healthy rats. During SNMP, bile flow averaged about 10 μl/g liver. It seems likely that the lower bile flow is due to the lower temperature during SNMP. Bile production was not significantly higher in the Fresh-SNMP group than in the WI-SNMP group (Figure 5D).

### Posttransplantation blood analysis

**Figure 6:**
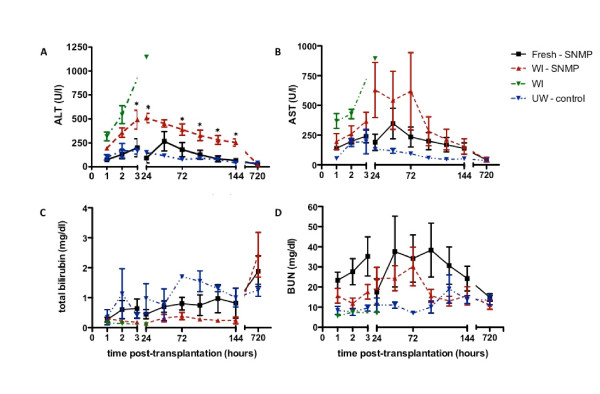
**Blood results post-transplantation included alanine aminotransferase (A), aspartate aminotransferase (B), total bilirubin (C) and blood urea nitrogen (D).** Error bars = SEM. *P < 0.05 between the WI-SNMP group and both the Fresh-SNMP and UW-Control groups.

Blood work was performed hourly for the first 3 hours, then daily for 7 days and finally 30 days after transplantation. The most relevant parameters include serum transaminases (ALT and AST), total bilirubin (TB) and blood urea nitrogen (BUN) (Figure 6). In the 3 hours following reperfusion, transaminases spiked, most prominently in the WI-SNMP group (Figures 6A and 6B). Over the next 7 days, ALT levels were significantly higher in the WI-SNMP group, whereas the ALT levels in the Fresh-SNMP and UW-Control groups remained slightly elevated. Levels of AST were higher in both the WI-SNMP and Fresh-SNMP groups compared to the UW-Control group. In the WI group, the levels of ALT and AST climbed until death within the first 24 hours after surgery (Figures 6A and 6B). The nonsurviving animal in the WI-SNMP group displayed rapidly increasing levels of both AST and ALT, with readings at *t* = 72 hours in excess of 1,000 U/L. Among the surviving animals in all groups, the levels of AST and ALT were within normal range (<80 U/L) after the 30-day period.

TB increased mildly over the first 7 days, which was more apparent in the Fresh-SNMP group (Figure 6C). At the end of the 30-day period, TB was increased to approximately 2 mg/dl in all surviving animals. BUN levels were stable during the postoperative phase and throughout the 30-day period (Figure 6D).

**Figure 7:**
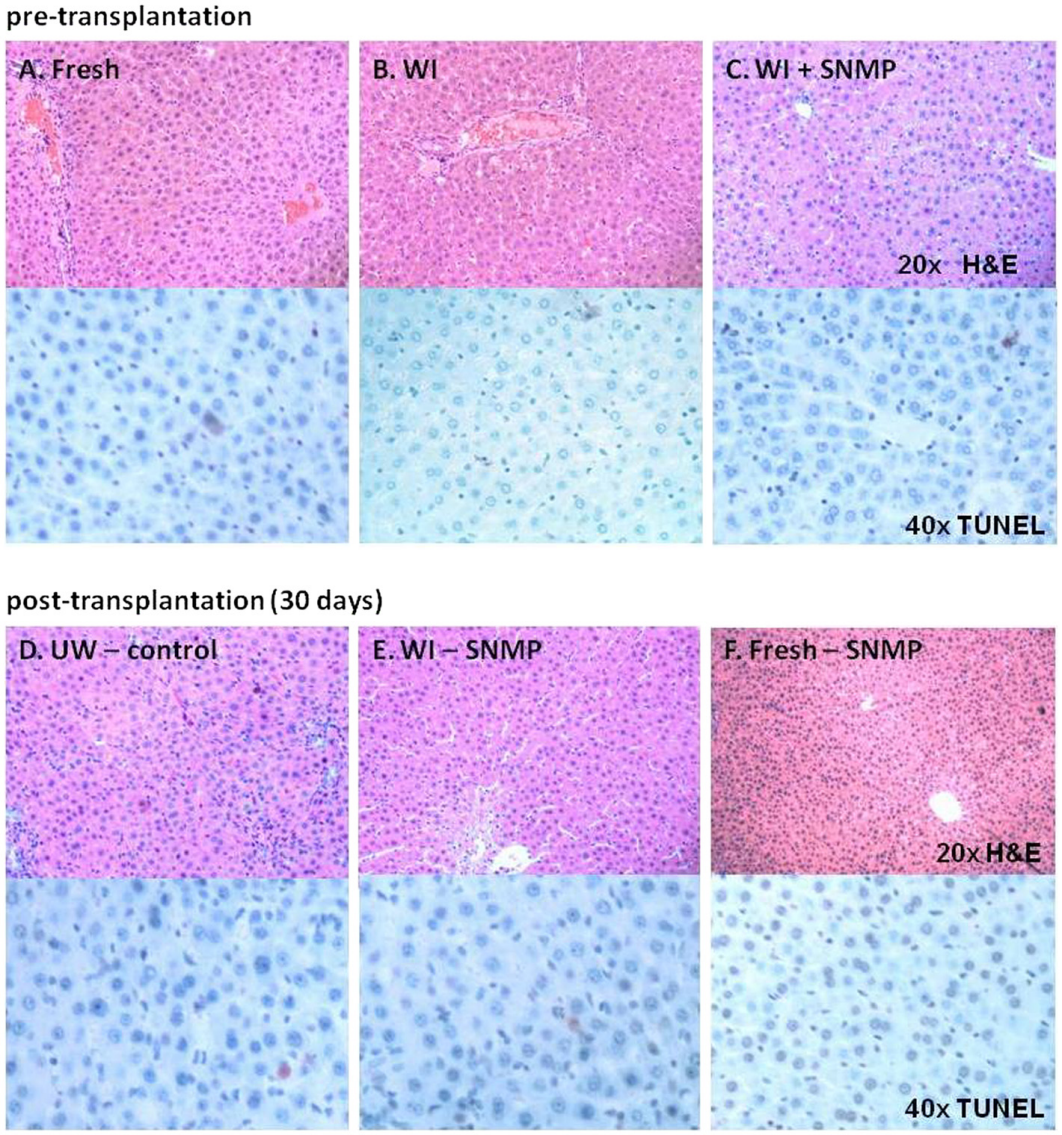
**Histological appearance of liver grafts pre- and posttransplantation (H & E staining and terminal deoxynucleotidyltransferase 2′-deoxyuridine 5′-triphosphate nick-end labeling (TUNEL) staining, respectively).** Top: sections from fresh liver, University of Wisconsin (UW) solution group and WI-SNMP groups. Bottom: 30 days posttransplantation autopsic resections from all survivors (UW-Control group, Fresh-SNMP and WI-SNMP groups).

Histological analysis (H & E and TUNEL staining) of livers pre- and posttransplantation is displayed in Figure 7. Although a few apoptotic cells were seen in all of the samples, including fresh liver (Figure 7A), there were no signs of necrosis or cellular swelling, with the exception of the WI group (Figure 7B). Pyknotic cells were observed throughout this sample, indicating cellular degeneration. After 3 hours of SNMP (WI-SNMP group) (Figure 7C), this finding disappeared, so that the tissue histomorphologically resembled fresh liver tissue. At 30 days posttransplantation, all groups showed normal hepatocellular architecture and microvasculature (Figures 7D to 7F). Hyperplasia of biliary epithelium was observed in all transplanted livers, inherent to this model, which uses a stent for the bile duct anastomosis.

## Discussion

SCS is practical and cost-effective but does not allow the resuscitation of damaged grafts, which is vital to the extended incorporation of marginal organs [43]. In addition, evaluation of organ quality during SCS is limited to subjective assessments. To combine the simplicity of SCS with the therapeutic impact of machine perfusion, we established a practical SNMP system that requires no temperature control, donor blood or artificial oxygen carriers. In an analogous study by our group, this system was used to optimize livers for hepatocyte isolation (ML Izamis, personal communication). Herein we have shown that fresh livers could be preserved for 3 hours and transplanted successfully, with survival rates and secondary outcomes equivalent to conventional cold storage parameters. Furthermore, we used this system to treat livers with sustained warm ischemia, which were then successfully transplanted.

In the animal from the WI-SNMP group that died within 72 hours after transplantation, the highly elevated levels of AST and ALT point to primary nonfunction. During treatment, the portal pressure of this liver was significantly increased, suggesting a correlation between high vascular resistance and graft failure. Although this is a singular finding in our study, the principle of vascular resistance as a predictor of graft viability has been suggested elsewhere [44]. We conclude that high vascular resistance during machine perfusion could serve as a warning sign indicative of a mechanical or physiological problem. However, specific research into this phenomenon should be continued on a broad scale.

Cumulative ALT levels during SNMP were highest in the WI-SNMP group. After 1 hour of SNMP, these levels reached a plateau, signifying that the hepatocyte damage caused by the WI period had been contained. Warm ischemic and fresh livers consumed similar amounts of oxygen during the SNMP period. We observed that oxygen consumption rates were not affected by the organ’s condition. We can conclude that resuscitation of a warm ischemic liver to a transplantable state does not require more oxygen than the SNMP preservation of a fresh liver. In addition, the high residual oxygen content in the outflow perfusate indicates that none of the livers exhausted the supply of oxygen. Moreover, the ATP recovery of warm ischemic livers beyond fresh levels also points to adequate aerobic respiration in the ischemic grafts. Thus we conclude that our system did not require an oxygen carrier.

The WI-SNMP group showed higher levels of transaminase leakage posttransplantation, indicating that although the livers were reclaimed adequately for recipient survival, there was still regenerative potential that remained unfulfilled. These observations are concurrent with the body weight trends: animals in all groups initially lost weight, but the UW-Control and Fresh-SNMP groups regained that weight sooner than the WI-SNMP group. Possibly, SNMP was responsible for only part of the restoration process and the remainder was carried out at the expense of the recipient. Another explanation may be that certain specific effects of warm ischemia were not mitigated by the SNMP and could be only restored by the recipients themselves, which in this case were young and healthy animals. As rats have great regenerative capacity, this phenomenon may be of greater consequence in a higher-order species. Although patients with end-stage liver disease are often in a fragile physical state, it has been shown that human livers from patients with elevated transaminases [45] or sustained warm ischemia [46] can be successfully transplanted. Whether our findings call for a longer or enhanced SNMP period will be clarified by continued investigation.

## Conclusion

In this study, we have established a protocol for SNMP, without oxygen carriers and without temperature control, that uses oxygenated and supplemented cell culture medium as perfusate. We applied this system to demonstrate feasibility against the current gold standard for the preservation of fresh organs, as well as the potential for regeneration of DCD grafts.

## Abbreviations

H & E: Hematoxylin and eosin; PBS: Phosphate-buffered saline.

## Competing interests

The authors declare that no conflicts of interest exist in connection with this manuscript.

## Authors’ contributions

TAB participated in conceiving the study, designing the experiments, conducting the study and writing the manuscript. BGB participated in conducting research and writing the manuscript. JL participated in collecting the data. VdA participated in collecting the data. QL performed the statistical analysis and participated in data collection. MLI participated in conceiving the study, designing the experiments and writing the manuscript. KU participated in conceiving the study and designing the experiments and edited the manuscript. MLY participated in conceiving the study and designing the experiments. All authors read and approved the final manuscript.

## References

[B1] Human Resources and Services Administration **Organ Procurement and Transplantation Network and the Scientific Registry of Transplant Recipients: 2009 OPTN/SRTR Annual Report: Transplant Data 1999–2008** [http://optn.transplant.hrsa.gov/ar2009/]

[B2] Schleifer D A simple heart-lung machine for the perfusion of small laboratory animals and for organ perfusion] [in German Chirurg 1967 38 477 480 5593492

[B3] Guarrera JV Henry SD Samstein B Odeh-Ramadan R Kinkhabwala M Goldstein MJ Ratner LE Renz JF Lee HT Brown RS Jr Emond JC Hypothermic machine preservation in human liver transplantation: the first clinical series Am J Transplant 2010 10 372 381 10.1111/j.1600-6143.2009.02932.x 19958323

[B4] Xu H Lee CY Clemens MG Zhang JX Prolonged hypothermic machine perfusion preserves hepatocellular function but potentiates endothelial cell dysfunction in rat livers Transplantation 2004 77 1676 1682 10.1097/01.TP.0000129644.23075.71 15201666

[B5] Lee CY Jain S Duncan HM Zhang JX Jones JW Jr Southard JH Clemens MG Survival transplantation of preserved non-heart-beating donor rat livers: preservation by hypothermic machine perfusion Transplantation 2003 76 1432 1436 10.1097/01.TP.0000088674.23805.0F 14657681

[B6] Lee CY Zhang JX Jones JW Jr Southard JH Clemens MG Functional recovery of preserved livers following warm ischemia: improvement by machine perfusion preservation Transplantation 2002 74 944 951 10.1097/00007890-200210150-00008 12394835

[B7] Jain S Xu H Duncan H Jones JW Jr Zhang JX Clemens MG Lee CY Ex-vivo study of flow dynamics and endothelial cell structure during extended hypothermic machine perfusion preservation of livers Cryobiology 2004 48 322 332 10.1016/j.cryobiol.2004.01.010 15157780

[B8] Saad S Minor T Short-term resuscitation of predamaged donor livers by brief machine perfusion: the influence of temperature Transplant Proc 2008 40 3321 3326 10.1016/j.transproceed.2008.06.058 19100381

[B9] Taylor MJ Baicu S Leman B Greene E Vazquez A Brassil J Twenty-four hour hypothermic machine perfusion preservation of porcine pancreas facilitates processing for islet isolation Transplant Proc 2008 40 480 482 10.1016/j.transproceed.2008.01.004 18374108 PMC2413169

[B10] Jain S Lee SH Korneszczuk K Culberson CR Southard JH Berthiaume F Zhang JX Clemens MG Lee CY Improved preservation of warm ischemic livers by hypothermic machine perfusion with supplemented University of Wisconsin solution J Invest Surg 2008 21 83 91 10.1080/08941930701883657 18340625

[B11] Vogel T Brockmann JG Friend PJ Ex-vivo normothermic liver perfusion: an update Curr Opin Organ Transplant 2011 15 167 172 20186059 10.1097/MOT.0b013e328337349d

[B12] Perkins JD Defatting the fatty liver with normothermic perfusion of the liver allograft Liver Transpl 2009 15 1366 1367 19806685

[B13] Brockmann J Reddy S Coussios C Pigott D Guirriero D Hughes D Morovat A Roy D Winter L Friend PJ Normothermic perfusion: a new paradigm for organ preservation Ann Surg 2009 250 1 6 10.1097/SLA.0b013e3181a63c10 19561463

[B14] Reddy SP Brockmann J Friend PJ Normothermic perfusion: a mini-review Transplantation 2009 87 631 632 10.1097/TP.0b013e3181995e83 19295304 PMC5842890

[B15] Mann CD Metcalfe MS Nicholson ML Normothermic perfusion of ischaemic porcine kidneys: an evaluation of ex vivo function and endothelin receptor antagonism J Nephrol 2009 22 144 151 19229830

[B16] Tolboom H Pouw RE Izamis ML Milwid JM Sharma N Soto-Gutierrez A Nahmias Y Uygun K Berthiaume F Yarmush ML Recovery of warm ischemic rat liver grafts by normothermic extracorporeal perfusion Transplantation 2009 87 170 177 10.1097/TP.0b013e318192df6b 19155970 PMC2743395

[B17] Tolboom H Milwid JM Izamis ML Uygun K Berthiaume F Yarmush ML Sequential cold storage and normothermic perfusion of the ischemic rat liver Transplant Proc 2008 40 1306 1309 10.1016/j.transproceed.2008.03.100 18589093 PMC2583139

[B18] Uygun K Tolboom H Izamis ML Uygun B Sharma N Yagi H Soto-Gutierrez A Hertl M Berthiaume F Yarmush ML Diluted blood reperfusion as a model for transplantation of ischemic rat livers: alanine aminotransferase is a direct indicator of viability Transplant Proc 2010 42 2463 2467 10.1016/j.transproceed.2010.04.037 20832525 PMC3020900

[B19] Uemura R Uchiyama K Ozawa S Yamaue H Effect of normothermic perfusion using fructose-1,6-bisphosphate for maintenance of liver function duringin situextended hepatectomy by the total hepatic vascular exclusion technique J Surg Res 2007 137 89 95 10.1016/j.jss.2006.07.029 17084408

[B20] Imber CJ St Peter SD Lopez de Cenarruzabeitia I Pigott D James T Taylor R McGuire J Hughes D Butler A Rees M Friend PJ Advantages of normothermic perfusion over cold storage in liver preservation Transplantation 2002 73 701 709 10.1097/00007890-200203150-00008 11907414

[B21] Schön MR Kollmar O Wolf S Schrem H Matthes M Akkoc N Schnoy NC Neuhaus P Liver transplantation after organ preservation with normothermic extracorporeal perfusion Ann Surg 2001 233 114 123 10.1097/00000658-200101000-00017 11141233 PMC1421174

[B22] Kobayashi J Ohwada S Takeyoshi I Ohya T Tomizawa N Kamoshita N Kawashima Y Matsumoto K Morishita Y Normothermic perfusion using diluted blood ameliorates ischemia-reperfusion injury on the canine liver Transplant Proc 1998 30 3761 3762 10.1016/S0041-1345(98)01224-X 9838647

[B23] Hellinger A Fiegen R Lange R Rauen U Schmidt U Hirche H Kaiser S de Groot H Erhard J Eigler FW Preservation of pig liver allografts after warm ischemia: normothermic perfusion versus cold storage Langenbecks Arch Chir 1997 382 175 184 9395999 10.1007/BF02391863

[B24] Filipponi F Bacci S Romagnoli P Normothermic liver perfusion ex situ: a resuscitation tool for hepatic grafts damaged by warm ischemia G Chir 1993 14 254 258 8343355

[B25] Bond M Pitt M Akoh J Moxham T Hoyle M Anderson R The effectiveness and cost-effectiveness of methods of storing donated kidneys from deceased donors: a systematic review and economic model Health Technol Assess 2009 13 iii-iv, xi-xiv, 1 156 10.3310/hta13380 19674537

[B26] Belzer FO Southard JH The future of kidney preservation Transplantation 1980 30 161 165 10.1097/00007890-198009000-00001 14582169

[B27] Matias JE Morais FA Kato DM Koziak V Brioschi ML Tambara EM Agulham MÂ Coelho JC Prevention of normothermic hepatic ischemia during in situ liver perfusion with three different preservation solutions: experimental analysis by realtime infrared radiation thermography] [in Portuguese Rev Col Bras Cir 2010 37 211 217 21079894 10.1590/s0100-69912010000300009

[B28] Plauth M Zimmermann B Raible A Vieillard-Baron D Bauder-Gross D Hartmann F Use of an artificial oxygen carrier in isolated rat liver perfusion: first demonstration of net glucose uptake at physiological portal glucose concentrations using a hemoglobin-free perfusate Res Exp Med (Berl) 1991 191 339 347 10.1007/BF02576689 1759045

[B29] Vairetti M Ferrigno A Carlucci F Tabucchi A Rizzo V Boncompagni E Neri D Gringeri E Freitas I Cillo U Subnormothermic machine perfusion protects steatotic livers against preservation injury: a potential for donor pool increase? Liver Transpl 2009 15 20 29 10.1002/lt.21581 19109848

[B30] Vairetti M Ferrigno A Rizzo V Boncompagni E Carraro A Gringeri E Milanesi G Barni S Freitas I Cillo U Correlation between the liver temperature employed during machine perfusion and reperfusion damage: role of Ca^2+^ Liver Transpl 2008 14 494 503 10.1002/lt.21421 18383108

[B31] Gringeri E Polacco M D’Amico FE Scopelliti M Bassi D Bonsignore P Luisetto R Lodo E Carraro A Zanus G Cillo U A new liver autotransplantation technique using subnormothermic machine perfusion for organ preservation in a porcine model Transplant Proc 2011 43 997 1000 10.1016/j.transproceed.2011.01.139 21620035

[B32] Tolboom H Izamis ML Sharma N Milwid JM Uygun B Berthiaume F Uygun K Yarmush ML Subnormothermic machine perfusion at both 20°C and 30°C recovers ischemic rat livers for successful transplantation J Surg Resin press 10.1016/j.jss.2011.03.003 PMC3863393 21550058

[B33] Muriel A López V Zamora Vincente J Gutiérrez C Abraira Santos V Hernández Marrero D [Does hypothermic machine perfusion provide an advantage over cold storage in the incidence rate of delayed graft function following a deceased-donor kidney transplant?] [in Spanish] Nefrologia 2009 29 6 Suppl 85 87 20221238 10.3265/NEFROLOGIA.2009.29.S.E.noID.36.free

[B34] Delrivière L Gibbs P Kobayashi E Goto S Kamada N Gianello P Technical details for safer venous and biliary anastomoses for liver transplantation in the rat Microsurgery 1998 18 12 18 10.1002/(SICI)1098-2752(1998)18:1<12::AID-MICR4>3.0.CO;2-W 9635788

[B35] Dutkowski P Furrer K Tian Y Graf R Clavien PA Novel short-term hypothermic oxygenated perfusion (HOPE) system prevents injury in rat liver graft from non-heart beating donor Ann Surg 2006 244 968 977 10.1097/01.sla.0000247056.85590.6b 17122622 PMC1856639

[B36] Tolboom H Pouw R Uygun K Tanimura Y Izamis ML Berthiaume F Yarmush ML A model for normothermic preservation of the rat liver Tissue Eng 2007 13 2143 2151 10.1089/ten.2007.0101 17596120

[B37] Kamada N Calne RY Orthotopic liver transplantation in the rat: technique using cuff for portal vein anastomosis and biliary drainage Transplantation 1979 28 47 50 10.1097/00007890-197907000-00011 377595

[B38] Mabuchi A Wake K Marlini M Watanabe H Wheatley AM Protection by glycyrrhizin against warm ischemia-reperfusion-induced cellular injury and derangement of the microcirculatory blood flow in the rat liver Microcirculation 2009 16 364 376 10.1080/10739680902796917 19308793

[B39] Szijártó A Methods of increasing ischemic tolerance in liver surgery] [in Hungarian Magy Seb 2008 61 128 135 10.1556/MaSeb.61.2008.3.5 18515218

[B40] Izamis ML Sharma NS Uygun B Bieganski R Saeidi N Nahmias Y Uygun K Yarmush ML Berthiaume F In situ metabolic flux analysis to quantify the liver metabolic response to experimental burn injury Biotechnol Bioeng 2011 108 839 852 10.1002/bit.22998 21404258 PMC3277812

[B41] Olschewski P Gass P Ariyakhagorn V Jasse K Hunold G Menzel M Schöning W Schmitz V Neuhaus P Puhl G The influence of storage temperature during machine perfusion on preservation quality of marginal donor livers Cryobiology 2011 60 337 343 20233587 10.1016/j.cryobiol.2010.03.005

[B42] Sumimoto K Inagaki K Yamada K Kawasaki T Dohi K Reliable indices for the determination of viability of grafted liver immediately after orthotopic transplantation: bile flow rate and cellular adenosine triphosphate level Transplantation 1988 46 506 509 10.1097/00007890-198810000-00007 3051557

[B43] García-Valdecasas JC Fondevila C In-vivo normothermic recirculation: an update Curr Opin Organ Transplant 2010 15 173 176 10.1097/MOT.0b013e3283373488 20186060

[B44] Monbaliu D Vekemans K De Vos R Brassil J Heedfeld V Qiang L D’Hollander M Roskams T Pirenne J Hemodynamic, biochemical, and morphological characteristics during preservation of normal porcine livers by hypothermic machine perfusion Transplant Proc 2007 39 2652 2658 10.1016/j.transproceed.2007.08.009 17954200

[B45] Radunz S Paul A Nowak K Treckmann JW Saner FH Mathé Z Liver transplantation using donor organs with markedly elevated liver enzymes: how far can we go? Liver Int 2011 31 1021 1027 10.1111/j.1478-3231.2011.02525.x 21733092

[B46] Monbaliu D Pirenne J Talbot D Liver transplantation using donation after cardiac death donors J Hepatol 2012 56 474 485 21782762 10.1016/j.jhep.2011.07.004

